# 3D Printed Patient-Specific Complex Hip Arthroplasty Models Streamline the Preoperative Surgical Workflow: A Pilot Study

**DOI:** 10.3389/fsurg.2021.687379

**Published:** 2021-08-26

**Authors:** Michael Jiang, Jasamine Coles-Black, Gordon Chen, Matthew Alexander, Jason Chuen, Andrew Hardidge

**Affiliations:** ^1^3dMedLab, Austin Health, The University of Melbourne, Parkville, VIC, Australia; ^2^Department of Surgery, Austin Health, The University of Melbourne, Heidelberg, VIC, Australia

**Keywords:** 3D printing, orthopaedic surgery, simulation, presurgical planning, healthcare systems

## Abstract

**Introduction:** Surgical planning for complex total hip arthroplasty (THA) often presents a challenge. Definitive plans can be difficult to decide upon, requiring unnecessary equipment to be ordered and a long theatre list booked. We present a pilot study utilising patient-specific 3D printed models as a method of streamlining the pre-operative planning process.

**Methods:** Complex patients presenting for THA were referred to the research team. Patient-specific 3D models were created from routine Computed Tomography (CT) imaging. Simulated surgery was performed to guide prosthesis selection, sizing and the surgical plan.

**Results:** Seven patients were referred for this pilot study, presenting with complex conditions with atypical anatomy. Surgical plans provided by the 3D models were more detailed and accurate when compared to 2D CT and X ray imaging. Streamlined equipment selection was of great benefit, with augments avoided post simulation in three cases. The ability to tackle complex surgical problems outside of the operating theatre also flagged potential complications, while also providing teaching opportunities in a low risk environment.

**Conclusion:** This study demonstrated that 3D printed models can improve the surgical plan and streamline operative logistics. Further studies investigating the optimal 3D printing material and workflow, along with cost-benefit analyses are required before this process is ready for routine use.

## Introduction

Total hip arthroplasty (THA) has been a highly successful operation worldwide since its inception (1). The main indications for the procedure are pathologies which alter the biomechanics of the hip joint: most commonly osteoarthritis, fracture, and tumour infiltration. These conditions displace the centre of rotation of the joint via bony destruction. THA aims to correct these defects by restoring the centre of rotation, maintaining alignment and offset of the joint, preserving adequate bone stock and ensuring stability of the hip joint through either a cemented or uncemented prosthesis (2). Uncemented acetabular prostheses require ~50–60% surface area coverage and two thirds rim fit to provide adequate fixation for native bone to heal into and create union (3). The optimal position for an uncemented prosthesis requires both sufficient fixation and orientation, with 6 degrees of freedom in which errors can occur (4). In patients with atypical anatomy, this can be very challenging to achieve.

In routine THA, the size and position of the required implants is optimised using templating X rays (XR) (5). In patients with atypical or disrupted acetabular anatomy, more extensive investigation is necessary (6). Computed tomography (CT) is used in these cases to image the relevant anatomy in three-dimensional space; however preoperative planning based on CT alone is often insufficient to decide upon a definitive procedure. Multiple surgical plans must be prepared, leading to an increased workload for the surgical team, along with increased logistical and financial burden. 3D printed patient-specific models for preoperative planning have been suggested as an approach for these complex cases, and have demonstrated clinical benefit in this patient cohort (7).

Previous studies regarding the use of 3D models in surgical planning noted intraoperative benefits of reduced theatre time, decreased blood loss and shorter fluoroscopy time (7). Most studies shared a similar workflow, using preoperative CT imaging to create a digital render which was transferred to a 3D printer for model creation (8–12). Models were used for anatomical appraisal of relevant surgical anatomy, simulated surgery and templating of implants. Some studies also sterilised the 3D prints to be used in the intraoperative field as a reference to better orient the surgical anatomy, with this process being possible with inexpensive materials such as polylactic acid (PLA) which was able to be sterilised without deformity using high pressure steam (13–15). Consistently in the studies, surgeons felt that the 3D models were particularly useful in complex cases. Chen et al. (16) noted that visualisation of atypical anatomy alone was of benefit in planning the approach, with simulation and implant templating adding to the utility of the procedure. Bizzotto et al. (13) reported similar findings with 3D printed models being most useful for complex intra articular fractures with intra-articular steps of 2 mm or more.

While other methods such patient-specific instruments, custom 3D printed implants and computer aided preoperative planning have also been reported in the literature, the barrier to access with regards to initial investment is much greater (17, 18). In this pilot study, the authors present our initial experiences with 3D printed patient-specific models produced in-house with open source software for pre-surgical planning. We describe how models have improved surgical planning and the perioperative workflow in complex THA.

## Methods

### Patient Selection

Patients included in this study were those requiring THA with challenging surgical anatomy from July 2018 to December 2019 at Austin Health, Melbourne, Australia. Inclusion criteria included complex anatomy which was difficult to appreciate through CT reconstruction alone. Conditions included complex pelvic fractures, osteoarthritis complicated by substantial bone loss and patients with Perthes disease or developmental hip dysplasia. Suitable patients were referred to the research team by the orthopaedic unit at Austin Health. This study was approved by the Austin Health Human Research Ethics Committee in accordance with its guidelines. Informed consent was obtained from all patients when consenting for their surgical procedure.

### Medical Image Processing and Printing

Following routine preoperative CT, raw medical imaging data was processed using soft fines and bone fines algorithms, under guidance from the radiology department, and exported as Digital Imaging and Communications in Medicine (DICOM) files. Scans were performed using a GE Revolution CT scanner (Milwaukee, WI, USA) with 0.625 mm slice thickness, 100–120 kVp, modulated current of 300–600 mA and 30–40 cm FOV. A virtual 3D model of the patient anatomy was created using 3D Slicer (version 4.9; Harvard, US, 2019) (19), an open source medical image processing software. The workflow involved selectively including voxels above the 200–250 Hounsfield Unit (HDU) range, under advice from the radiology department at Austin Health, as this value was the lowest possible to reliably delineate bony anatomy from soft tissue while preserving cancellous bone architecture from the scan. Manual deletion of the femur was performed to define the acetabulum. Meshmixer (version 3.5; California, US, 2019), an open source computer aided design software, was used to repair mesh defects, remove extraneous surfaces and down-mesh the model to reduce file size prior to printing. Average processing time from acquiring the DICOM data to the completed Standard Triangle Language (STL) file suitable for 3D printing was 1 hour over the length of the study, with it being reduced to as little as 30 min by case 7. No difference in processing time was attributed to complexity of the case. Members of the orthopaedic team performed processing of digital images with technical input regarding printing parameters provided by the university engineering laboratory affiliated with the study. Previous studies have validated the dimensional accuracy of models created using this technique (15, 16, 20).

Completed STL files were 3D printed within 24 h, using a variety of materials as described in Table 1. The first three cases were created using VeroWhite resin (Stratasys, Eden Prairie, MN, USA), with the following three created from plaster. The final case utilised all three materials and was the basis for the material comparison.

**Table 1:** A summary of the three materials trialled for 3D printed patient-specific models and cost per model.

**Material**	**3D Printer**	**Average cost per model (US$)**
Plaster	Projet 660 (3D Systems Corporation, Rock Hill, SC, USA)	200
VeroWhite Resin	Objet 30 (Stratasys, Eden Prairie, MN, USA)	1,500
Nylon	Jet Fusion 4200 (Hewlett-Packard, Paola Alto, CA, USA)	100

### Simulated Surgery

Surgical simulation was performed by the consultant and registrar responsible for each case. Each model was placed on a theatre tray, fixed in the position expected for a posterior approach, with a routine THA instrumentation set up prepared for reaming. The consultant and registrar then reamed the acetabulum in successive increments replicating the intraoperative process. Reaming was attempted to the appropriate size, with some models reamed further to test for acetabular wall integrity while aiming to maximise rim fit. Templating of the cup was based on the seating of the implant, rim fit and bone stock in the surrounding walls post reaming. Post reaming, a trial cup was secured and impacted where possible to assess for fixation. Finally, further implants such as plates, augments and cages were trialled to demonstrate suitability and templated where required. The femoral side was unable to be templated as all materials used in this study deformed to an unacceptable level upon impaction.

Simulation on average required 15 min for a straightforward case with one type of material. Additional time was required for contouring of plates and when complications, such as fracture, were encountered. Following simulation, the surgeons recorded a surgical plan and estimated sizing of any prostheses required in the surgery, which was then compared to the data obtained from planning using templating XRs.

## Results

Seven patients underwent complex THA during the study period, using 3D printed models as an adjunct to pre-surgical planning (Table 2). Patient-specific models were 3D printed in plaster, resin, and nylon.

**Table 2:** A summary of the cases involved in this pilot study including demographic data, causative pathology leading to THA and changes to surgical management.

				**Templated size of acetabular cup (mm)**				**Material**	**Notes on image processing**	**Cost (USD)**
**Case**	**Age**	**Gender**	**Pathology**	**2D**	**3D**	**Intra-operative**	**Changes to surgical plan**			
1	92	F	Acetabular fracture	48	48	52	On simulation, it was found that there was an undiscovered fracture of the posterior ramus which fractured on reaming. The decision was made to pre counter a plate and sterilise it for moulding on the back table to stabilise the fracture. Trialling also indicated the patient was not suitable for a cage, therefore one was not ordered. A femoral head graft was opted for to fill the posterior wall defect. On reaming the cortical edges spun dangerously, and the decision was made to cut the femur directly under the head to ream.	VeroWhite	This model provided an acceptable representation of patient anatomy. The support material provided an analogue to the soft tissue and cancellous bone. Reaming and impaction was well supported. The fracture pattern was well preserved by the support material. Processing time of the model was 1.5 hrs	1,600
2	52	M	Perthes' disease	54	52	52	When viewing the CT imaging, augments were decided upon. On simulation it was determined that augments were not necessary and that adequate fixation was able to be achieved. Augments were not ordered.	Verowhite	This model provided an accurate representation of patient anatomy, with adequate assessment of fixation. Not difficulties were encountered on reaming. Processing time for this model was 1.5 hrs	1,500
3	53	M	Perthes' disease	46	60	50	This patient was trialled with the Stryker RAS system which included an augment within the acetabular cup itself, hence the large difference in templating size. Augments were considered for this case however intraoperatively there was adequate fixation with the superior edge uncovered.	Verowhite	The resin model was able to withstand reaming using the oversized RAS system, however impaction was not satisfactory with a poor rim fit resulting in a unsatisfactory simulation of cup fit. Processing time of the model was 1 h	1,550
4	89	M	Acetabular fracture	64	60	62	The patient had a complex acetabular fracture with anterior column discontinuity. Augments and a cup/cage complex were prepared for this case. Augments were trialled on the model and sized at 50 mm. The superior bone stock was deemed adequate for screws on visualisation of the model. Intraoperatively, the superior screws provided adequate fixation and other implants were not required.	Plaster	The fracture pattern printed using the plaster was quite frail. The posterior wall segment and anterior column discontinuity required additional construction as they both fell off post print. Processing time for the model was 1 h.	250
5	84	F	Severe osteoarthritis and femoral head necrosis	48	48	48	The anterior wall of this model was shown to be deficient on reaming. The decision was made to bias reaming posteriorly to preserve anterior bone stock. A 48 mm cup press fit in the model which was reflected intraoperatively.	Plaster	The plaster model in this case provided an accurate haptic mimic to bone. The acetabular wall had solid bone stock on CT and as such reaming was very realistic with an accurate representation of rim fit which was replicated intraoperatively. Processing time for this model was 1 hr	200
6	46	F	Severe osteoarthritis and femoral head necrosis	46	52	54	Patient presented with bilateral severe OA and femoral head necrosis. 2D templating proved difficult using the affected or contralateral acetabulum. 3D simulation was much more reflective of the intraoperative conditions.	Plaster	Reaming of this model required care due to the lack of support material within the acetabulum. The anterior wall was nearly breached and almost failed. Processing time for this model was 0.5 hrs.	200
7	50	M	Severe osteoarthritis and femoral head necrosis	54	58	58	2D templating was difficult as patient had considerable bone loss and was not comparable to the contralateral side. Augment was trialled. Intraoperatively there was adequate fixation with 3 screws.	Plaster, VeroWhite, Nylon	This case provided the material comparison noted in the material properties section. Image processing was 0.5 hrs with a longer simulation time to account for all models.	Resin: 1,200 Plaster: 250 Nylon: 100

*Templated size of implant on XR and CT (2D) vs. on the patient specific model (3D) is shown and compared to the size of definitive implant decided upon intraoperatively*.

Simulation with patient-specific 3D printed models conferred superior clinical, logistical and educational outcomes compared to CT and XR. Deliberate practice with the models prior to the operation streamlined equipment selection and revealed potential complications, allowing them to be accounted for intraoperatively. Ordering of equipment was able to be reduced to only the necessary trays, reducing the logistical and financial burden involved. Surgical simulation also provided a low-pressure environment for teaching without risk to the patient (21).

### Material Properties

**Figure 1:**
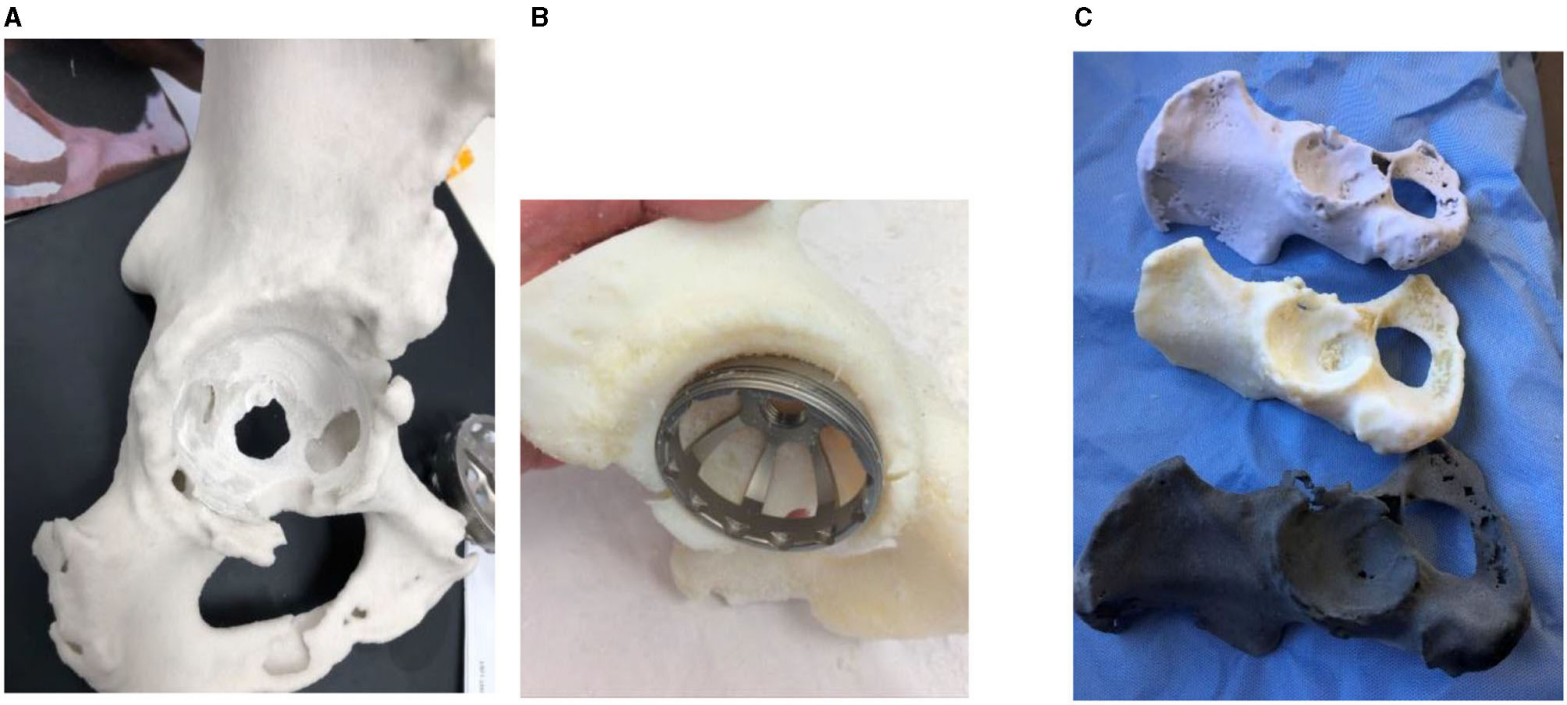
From left to right **(A)** The plaster models possessed the most realistic haptic feedback during simulated surgery, with each revolution of the reamer removing a layer of plaster which closely reflects what occurs intraoperatively. **(B)** The resin models performed best when simulating implant fixation due to the surrounding support material which mimicked soft tissue structures absent in other models. **(C)** A comparison of all three materials with the same model created in from top down plaster, resin and nylon.

Plaster performed best when reaming the models with the most realistic haptic feedback of the three materials (Figure 1). Plaster models are created by fusion of layers of plaster powder, thus allowing each cycle of the reamer to scrape away a small layer and most effectively reproduced the grasp of an intraoperative ream which was not reproduced by the resin or nylon. However, these models often had deficiencies in the surrounding acetabulum due to lack of bony detail within the cancellous bone on CT imaging. This rendered them prone to shattering if reamed too far past the acetabular shell of bone. In comparison, resin provided the most realistic trial of implant impaction due to the presence of support material which was left *in situ* in anatomical locations to approximate soft tissue. The support material in the resin models prevented this issue, and allowed impaction of the implant into the model without breakage. However, as the layers of resin and support are fused together, reaming was more strenuous. For the average hemi-pelvis printed in this study, each plaster model cost ~USD$200, while resin models were the most expensive at USD$1,500 including both the resin and the necessary support material. The costs of these models would be increased if a larger section of the pelvis was required. Nylon models were the most cost-effective at USD$100 per model, however its material properties were found to be least favourable on simulation, in accordance with findings from other studies (22). These models were prone to warping on reaming, and bony architecture quickly became distorted. Rotation of the reamer within the model resulted in rotational stretching of the layers within the model, thus losing anatomical accuracy.

## Discussion

Our pilot study reports encouraging findings indicating that simulation with patient-specific models narrows the definitive surgical plan, streamlines prosthesis selection and predicts potential complications prior to complex THA.

### Limitations

The authors acknowledge the limitations of this pilot study, due to the small cohort size. Furthermore, comparison to a control group is extremely challenging in a cohort of unique and complex cases, where even patients with the same condition often present with vastly different anatomy. Both these limitations are inherent to the nature of the pathologies addressed with complex THA. This dilemma has been raised previously by Karlin et al. (9) who commented on the difficulty in creating a satisfactory control group for complex pathologies with clinical heterogeneity within the same disease classification. In this study, more complex patients were enrolled into the 3D print group as planning would have been extremely challenging with conventional planning methods.

Longer term outcome data is also required in order reach a definitive conclusion on the benefits to patient safety and quality of life. As 3D printing technologies continue to improve, the methods for creating models requires further streamlining to ascertain the most appropriate material and printer type as well as integration into the wider surgical system.

### Clinical Benefits

Surgical simulation allowed us to trial multiple approaches to the same surgical problem. Patients recruited for this study provided unique challenges with complex atypical anatomy rendering traditional templating methods unreliable. Deliberate practice with patient specific anatomy provided the surgeon with key information including if an augment was required, whether a rim fit acetabular cup was adequate for fixation or if alternatives were required, and the size progression and orientation of the intraoperative ream achieved safely. A more confident approach into the acetabulum can also be made, with visualisation and simulation informing the surgeon of any potential obstructions from osteophyte or other bony prominence along with the knowledge of which of these can be safely resected to improve access without compromising fixation later on.

In cases of pelvic fracture, the model better visualised the fracture pattern and allowed all fragments to be accounted for intraoperatively, with the additional benefit of allowing trialling and pre-contouring of plates and screws required.

For example, Case 3 involved an acetabulum with 3 plane mismatch which would have likely caused blow out of the medial wall with a cup that could secure a rim fit in the acetabulum. Due to this, a smaller cup with augments was planned. On simulation, it was shown that a smaller cup was able to be secured with adequate fixation and no augmentation despite leaving the superior edge exposed. This avoided the increased operating time, equipment cost and potential for failure associated with the augment. Templating via this method is extremely valuable in these cases, as the affected side is often too disrupted to confidently template, with the contralateral side too dissimilar to use reliably.

Pre-contouring of implants was another valuable aspect when simulating complex THA with patient-specific models. Templating of plates, cages and screw placements with 3D printed models led to significant reductions in operating time, as reflected in the study by Chana-Rodriguez et al. in which a plate was able to be implanted intra-operatively in a case of complex acetabular fracture without adjustment post templating on a 3D printed model (23). This was reflected in the first case, with the patient presenting with a complex acetabular fracture and associated protrusio acetabuli. Upon reaming of the model, a fracture line previously thought insignificant on review in radiology meetings failed, causing a posterior ramus fracture. The decision was made to plate this prior to reaming to prevent this complication intraoperatively hence a lead plate was contoured using the 3D model. The model allowed trialling of multiple plate positions with the most optimal decided upon for the final fixation. This plate was then sterilised and used to fashion the definitive implant intraoperatively on the back table, while the fracture site was prepared. The implant was secured with minimal further adjustment.

Similarly, screw placements were able to be assessed for viable bone stock, as seen in Cases 2, 4, and 7. Future cases which require similar screw, plate, cage or augment constructs could see significant operative benefit from the use of models to prefabricate the required implants.

Our pilot study also allowed for the identification of other potential complications, allowing preparation of contingency plans. Aside from the pubic ramus fracture identified in Case 1, adjustments were made to the femoral head graft. Originally, a subcapital cut was chosen, planning for the head to be placed into the acetabulum and reamed into the posterior wall defect. However, there was significant cortical bone present which caught on reaming and started to spin dangerously. Therefore, the femoral head was cut further superiorly, allowing the graft to be safely reamed into the defect. 3D printed patient-specific models were invaluable in predicting these intraoperative difficulties ahead of time, preventing stressful situations in the operating theatre.

Replication of the intraoperative process creates an environment of known processes from a previously uncertain procedure. The operation becomes streamlined as the optimal alignment and positioning of the ream along with the fit and orientation of the implant is being recreated instead of discovered. Stress inducing questions such as if increasing the size of the ream will improve the fit or cause a wall blowout, which obstructing anatomy can be removed, and if a non-conventional fixation is sufficient or will cause impingement have already been answered, giving the surgeon the confidence to proceed with their predetermined plan. The reduction in stress related to operative uncertainty can also be communicated to the patient, informing them of the risks associated with their complex procedure and the steps taken to mitigate the complications. Our study found these factors positively impacted surgical preparation from both a clinician and patient perspective.

### Logistical Benefits

**Figure 2:**
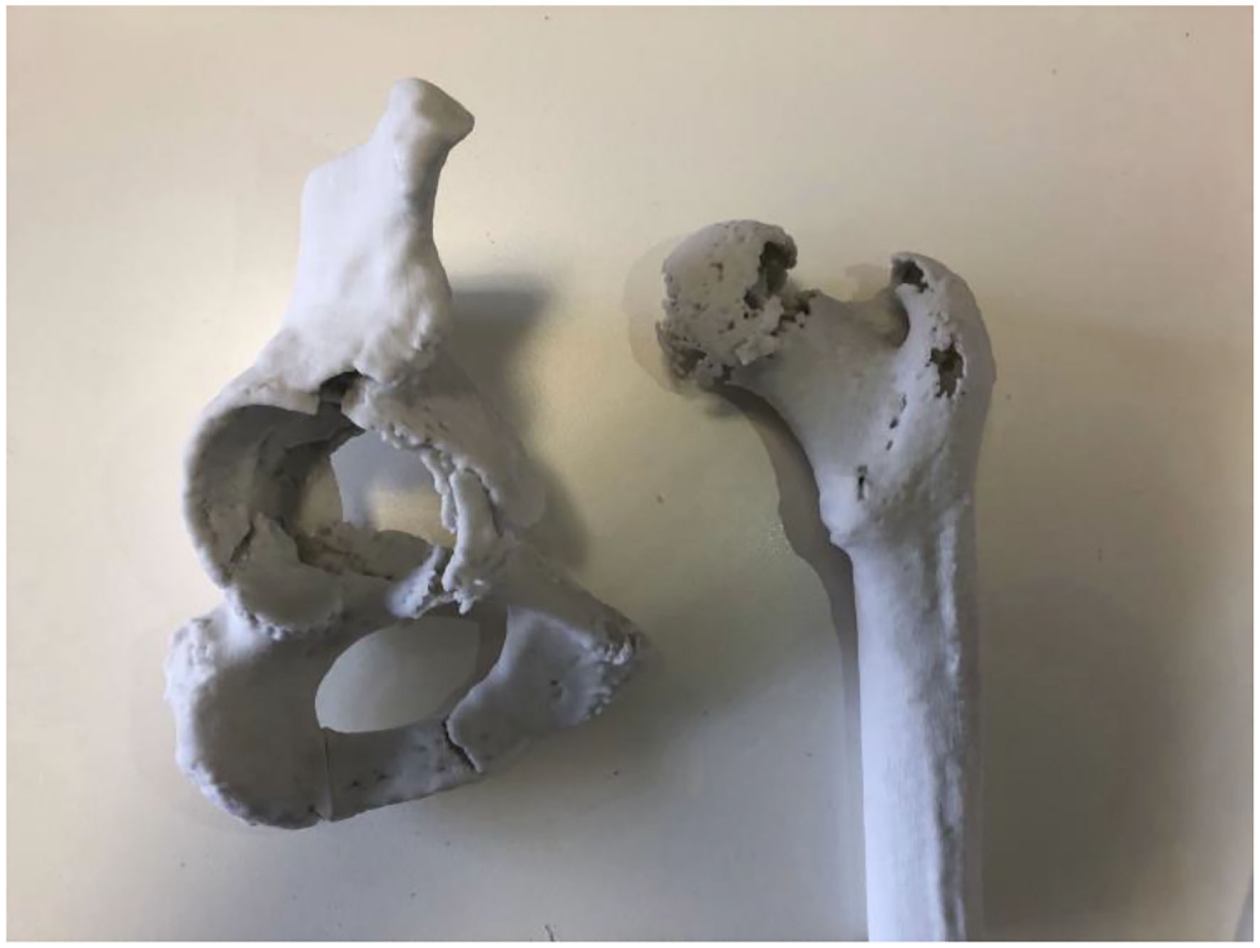
Plaster model from Case 4 showing an acetabular fracture with anterior column discontinuity. Despite the anterior column disruption it was shown that the posterior column was intact and stable, allowing for screws to be placed in the posterior and superior aspects of the cup to stabilise the construct without need for augments or cages.

Preparation for complex THAs involves the logistics of ordering, transport and sterilisation of all prostheses that may be required for the case. For complex cases, multiple sizes of acetabular cup, augments and cages are required in preparation for a definitive plan based on intraoperative findings. This equipment can comprise up to 14 trays for a standard THA with further equipment required for complex cases (24), conferring a significant logistical burden on the healthcare system. In addition, reducing the number of trays required in preparation would reduce the financial and environmental impact of the hospital. In our study, 3 cases which were originally planned for augments were shown to not require them post simulation. Simulation in Case 3 demonstrated that the smaller cup was able to be repositioned and medialised adequately such that augments were not required, with similar findings in Case 2. In Case 1, the femoral head was demonstrated as being suitable as a graft to fill the acetabular defect, again avoiding the need for augments. A further two cases were less definitive, with augments subsequently ordered but not used (Figure 2). Similar findings have been commented on in previous studies involving 3D modelling software in complex arthroplasty (25–27).

Reducing the unpredictability of complex cases also allows for theatre time to be allocated more efficiently. Difficult cases can be highly variable in the theatre time required, resulting in more conservative theatre bookings and staffing allocations. Simulation of complex cases provides greater clarity on the approach and techniques required, giving a more precise indication of case duration and allowing theatre bookings to be allocated more efficiently.

### Teaching

Although not initially a focus of our pilot study, it became apparent that simulating complex hip arthroplasty using 3D printed patient-specific models also provides a valuable teaching opportunity for trainees in a unique and low risk learning environment. Deliberate practice outside of the operating theatre allows the opportunity for trainees to plan, prepare and execute complex cases under the supervision of surgical educators while preserving patient safety. Due to this, simulated surgery using 3D models can not only provide a valuable tool in surgical planning but also a unique tool in surgical training (21).

### Workflow

This study aimed to demonstrate an example of a workflow from routine preoperative CT imaging to model creation that occurs entirely within a hospital environment for the planning of complex hip arthroplasty. Image processing and model creation was performed by members of the Orthopaedic team, with a rapid improvement in processing time noted from Cases 1 to 7. Once the initial learning curve had been overcome, cases could be processed in as little as 30 min. With utilisation of an in-house 3D printer, total turnaround was 24 h from scan to model. With use of open source software, this process could be integrated into a surgical unit with minimal outlay: primarily education of staff in the image processing procedure and the cost of the prints themselves.

In our experience, the decision to print in-house compared to a third party is influenced by cost and time. Third party production of the model is more expensive due to extra labour costs and has a longer turnaround time between scan and print. However, outsourcing the process eliminates the extra time requirement for the in-house staff and eliminates the set up and maintenance costs of housing the printer. Conversely, development of an in-house process can have drastically improved turnaround times. With a 24 h turnaround it may even be possible to apply this process to emergency trauma, which has been previously reported as unfeasible due to prolonged processing times (28). An in-house process in these cases could compress the time between scan, model creation and simulated surgery to one working day, while also providing greater input from the surgeon into the modelling process (29). This study also demonstrates that the learning curve can be quickly overcome by a surgical unit with minimal disruption to clinical workflow. The volume of models printed also weighs into the cost-benefit analysis, with set up and maintenance costs being less enticing if faced with a smaller case load (30).

While many options for segmentation and CAD software exist, the software selected in the study had the lowest barrier to access. Although lacking the automation and advanced features of some proprietary software, 3D slicer (version 4.9; Harvard, US, 2019) and Meshmixer (version 3.5; California, US, 2019) were sufficient for the manual segmentation and creation of 3D models of bony anatomy. If soft tissue structures were to be involved in further research, more advanced software would need to be considered to lessen the technical and time burden that would be associated. We encountered no patients with metalware *in situ*, and the resultant flare artefacts.

Regarding material selection, the ideal material would mimic the biomechanical properties of bone, while also allowing incorporation of surrounding soft tissues into the print. The haptic feedback would ideally mimic the grasp of the reamer as it removes layers of cortical and cancellous bone. However, this would need to be balanced against the brittleness of the material which would leave it prone to shattering when force was applied. In this pilot study, the plaster models reflected this best with the resin models providing additional resistance to what would be expected due to the fusion of layers of material. The material would also require sufficient viscoelasticity to allow testing for rim fit as the acetabular cups inserted are typically 1–2 mm greater in diameter than the reamer. In this aspect the resin was superior as the plaster was prone to shattering on impaction.

The current literature regarding the biomechanics of 3D printed materials mainly focuses on qualitative surgeon assessment of haptics, with quantitative studies still lacking (31). The femoral side was not investigated in this study due to material deficiencies. One femur was templated using resin however this model failed on impaction and was unable to withstand the forces necessary to hold the femoral prosthesis. While additional material could be used to reinforce the acetabular walls of the plaster models, this may confer extra strength not present within normal anatomy. As such, an ideal material combination still requires further research.

## Conclusion

Complex acetabular surgery continues to challenge orthopaedic surgeons, with new solutions and approaches continually emerging. This pilot study suggests that in-house creation of 3D printed patient-specific models can be rapidly integrated into a surgical unit, and can provide an array of benefits to the surgeon through the trialling of multiple approaches, devices and implants for complex THA, streamlining the logistics involved. In addition, they provided a unique teaching opportunity for surgical trainees.

This pilot study has informed our next steps to further streamline our workflow with regards to case selection, model creation and pre-operative rehearsals prior to the implementation of a larger-scale prospective trial. More broadly in the orthopaedic literature, further studies into the optimal printing workflow along with quantification of the financial benefits of the models are required before it can be justified for routine use.

## Data Availability Statement

The raw data supporting the conclusions of this article will be made available by the authors, without undue reservation.

## Ethics Statement

The studies involving human participants were reviewed and approved by Austin Health Department of Ethics. The patients/participants provided their written informed consent to participate in this study.

## Author Contributions

MJ, AH, and JC-B were involved in study conceptualisation and design. MJ and GC were involved in data collection. MJ, J-CB, AH, and MA were involved in manuscript writing. MA, GC, AH, and JC were involved in editing the final manuscript. All authors contributed to the article and approved the submitted version.

## Conflict of Interest

The authors declare that the research was conducted in the absence of any commercial or financial relationships that could be construed as a potential conflict of interest.

## Publisher's Note

All claims expressed in this article are solely those of the authors and do not necessarily represent those of their affiliated organizations, or those of the publisher, the editors and the reviewers. Any product that may be evaluated in this article, or claim that may be made by its manufacturer, is not guaranteed or endorsed by the publisher.
